# Normal pressure hydrocephalus: Neurophysiological and neuropsychological aspects: a narrative review

**DOI:** 10.1097/MD.0000000000028922

**Published:** 2022-03-04

**Authors:** Katia Micchia, Caterina Formica, Simona De Salvo, Nunzio Muscarà, Placido Bramanti, Fabrizia Caminiti, Silvia Marino, Francesco Corallo

**Affiliations:** 1 IRCCS Centro Neurolesi “Bonino-Pulejo”, S.S. 113, Via Palermo, C. da Casazza, 98124 Messina, Italy.

**Keywords:** dementia, evoked potentials, idiopathic normal pressure hydrocephalus, neurophysiology, neuropsychology

## Abstract

Idiopathic normal pressure hydrocephalus (NPH) is a syndrome that affects elderly people and is characterized by excessive accumulation of cerebrospinal fluid in the brain ventricles. Diagnosis is based on the evaluation of clinical symptoms, which consists of a classic triad (Hakim triad), gait disturbances, cognitive impairment, and urinary incontinence. However, this complete triad is not always seen; therefore, it is difficult to make the diagnosis. NPH can be divided into primary or idiopathic NPH and secondary NPH. Diagnostic criteria for NPH remain a topic of discussion; however, the development of diagnostic techniques has brought new opportunities for diagnosis. The aim of this review is to present an overview of neurophysiological and neuropsychological approaches to support the clinical evaluation of patients with NPH and contribute to the differential diagnosis of NPH and dementia, as the clinical symptoms of NPH may resemble other neurodegenerative disorders.

## 1 Introduction

Idiopathic normal pressure hydrocephalus (iNPH) is a syndrome that affects elderly people and is characterized by excessive accumulation of cerebrospinal fluid (CSF) in the brain ventricles. Despite typical ventricular enlargement, the pressure of the CSF remains normal and there is no history of an antecedent cause, such as severe head injury, subarachnoid hemorrhage, or tumor. This progressive pathology is responsible for a strongly disabling symptomatological picture.^[1]^ The diagnosis is based on the evaluation of clinical symptoms, which consists of a classic triad (Hakim triad), such as gait disturbances, cognitive impairment and urinary incontinence.^[2]^ However, this complete triad is not always seen; therefore, it is difficult to make the diagnosis.^[3]^ Normal pressure hydrocephalus (NPH) can be divided into primary or idiopathic NPH, where the underlying cause is not previously known and secondary NPH (sNPH), which can be the result of various pathologies such as subarachnoid hemorrhage, trauma, meningitis, and stroke.^[4,5]^ Marmarou et al^[6]^ argue that the combination of iNPH and sNPH cases can lead to considerable controversy in the diagnosis and therapy of such cases. To support the suspected diagnosis of iNPH, lumbar puncture, CT, and magnetic resonance imaging were performed with the aim of highlighting an increase in ventricular volume compared to cortical atrophy. The most commonly used treatment for NPH is the implantation of a ventricle–peritoneal (VP) shunt to drain the CSF from the cerebral ventricles to the peritoneum. A systematic review of 30 studies on VP shunt implants showed improvement in symptoms,^[7]^ but after the initial improvement, symptoms recurred despite evidence of shunt function. This has increased in older patients, and over time, the symptoms progress due to the neurodegenerative process.^[8]^ Therefore, identifying predictive factors for surgical outcomes is an urgent problem. Neuropsychological evaluation is a very useful diagnostic tool to evaluate neuropsychological deficits and neuropsychiatric disorders, providing evaluation criteria for early and differential diagnosis. Neurophysiological methods allow the recording of electrical activity in the brain. The use of various neurophysiological methods contributes to the topographical and functional diagnosis of the various anatomical structures involved in the lesion and provides information on the temporal changes induced by various pathologies. The aim of this review is to present an overview of neurophysiological and neuropsychological tests to support the clinical evaluation and the results obtained from the neuroimaging techniques of patients with NPH and to contribute to the differential diagnosis of NPH and dementia, as the clinical symptoms of NPH may resemble other neurodegenerative disorders.^[9]^

## 2 Methods

We conducted bibliographic research that was mainly based on the MEDLINE database of biomedical and PubMed scientific literature. We included a total of 41 articles. We used the following keywords “Normal pressure hydrocephalus and EEG” included 9 articles. ”Normal pressure hydrocephalus and auditory evoked potentials” included 2 articles. “Normal pressure hydrocephalus and motor evoked potentials” included 2 articles. ”Normal pressure hydrocephalus and related evoked potentials” included 3 articles. "Normal pressure hydrocephalus and somatosensory evoked potentials” included 1 article. For the research of neuropsychological and neuropsychiatric studies, research on PubMed database was conducted using the keywords “psychiatric symptoms in normal pressure hydrocephalus” included 9 articles. “Neuropsychology and normal pressure hydrocephalus” included 15 articles. We have considered both recent and less recent studies, due to the small number of articles about some aspects that we have treated, such as the psychiatric approach. We also looked at scientific citations on the Web of Science and excluded irrelevant studies. We excluded duplicates articles; no English articles and articles that was not focused on the aim of our review. The ethical approval from an ethics committee was not necessary because it was a review.

The ethical approval from an ethics committee was not necessary because it was a review.

## 3 Neurophysiological methods

### 3.1 Auditory evoked potentials

The measurement of evoked potentials has been applied as a noninvasive means to study hydrocephalus. The results suggest that serial measurement of evoked potentials in hydrocephalic patients may be useful in the management of hydrocephalus and in the evaluation of its influence on brain function. Clinical investigations of hydrocephalus through the use of auditory brainstem responses (ABR) have been conducted mainly in infants and children. McPherson et al^[10]^ noted a prolongation of the interpeak latency I to V, or central conduction time (CCT) in neonates with hydrocephalus, which normalized after the shunt. A study on ABR following subarachnoid hemorrhage used ABR to study the degree of brain damage in NPH and dementia and to predict the effectiveness of shunt surgery. The interpeak latencies of the ABR have been used as indices for assessing the function of the brain stem. Interpeak I to V was used as the central transmission time (CTT). The results showed that CTT was a reliable parameter of ABR, regardless of age. There was a significant prolongation of CTT in patients with NPH and the elderly dementia group compared to the other groups; however, there were no significant differences between them. There was some degree of brain stem dysfunction in patients with NPH and in patients with dementia, suggesting an aspect of the pathogenesis of NPH.^[11]^ ABRs were studied in 15 adults with suspected NPH before and after shunt placement. The patients were divided into groups A (shunt-ineffective) and B (shunt-effective) groups. The pre- and postoperative ABRs of each patient were compared with those of 20 normal volunteers and the relationships between ABR and some clinical outcomes. Preoperatively, 9 patients (60%) showed a prolonged CCT (interpeak latency of the I–V wave) compared to the mean control. The data suggest that abnormal CCT in suspected NPH may simply reflect the degree of brain stem involvement, since ABRs are sometimes normal when NPH is suspected. CCT was positively correlated with the preoperative clinical disability. These results suggest that brain dysfunction can be reversed by the shunt, but is not related to clinical disability in patients in whom the shunt is effective, that is, in patients with NPH. Measurement of ABRs appears to be a useful method for clinical monitoring of shunt patients.

### 3.2 Event related evoked potentials (ERP)

Event-related evoked potentials (ERPs) are a noninvasive method of monitoring physiological signal processing in relation to perception and memory functions in milliseconds on a time scale. Memory loss in NPH is often mild or moderate, but it can also be progressive and dominate the clinical picture. In contrast to the evolution of degenerative dementia, the course of NPH may be reversed by the patient's shunt. Clinical diagnostics do not provide sufficient information on the underlying AD pathology in patients with NPH. Distinguishing patients with NPH from those with AD can be difficult based on the clinical results. Recent studies^[12]^ have suggested that ERP recordings together with neuropsychological studies may predict a favorable outcome after the surgical procedure in patients with increased intracranial pressure. Savolainen et al^[13,14]^ compared automatic auditory ERPs, P50, N100, and MMN in patients with NPH and in patients with NPH and AD, which was verified by a brain biopsy obtained during the intraventricular intracranial insertion of a pressure catheter (ICP). According to the ERP results obtained, N100 and MMN can differentiate patients with dementia from those with increased intracranial pressure. The goal was to evaluate whether clear signs of AD can be detected in a simple way, using noninvasive neurophysiological measures before the intervention. These results suggest that ERPs are a useful method for differentiating pure NPH from NPH with concomitant AD.

### 3.3 Somato sensory evoked potentials (SEP)

Patients with sNPH do not always show the typical symptoms of dementia, gait abnormalities, and urinary incontinence, as in their idiopathic counterparts, and may experience other atypical symptoms, including seizures, impaired consciousness, and motor and sensory deficits. This is because primary diseases (SAH, head injury, intracranial tumors, meningitis, stroke, etc) often cause severe neurological deficits that could mask the typical symptoms of NPH and make it more difficult to establish a diagnosis.^[15]^ In a Danys et al^[16]^ examined 14 patients with NPH were examined using the somato sensory evoked potentials (SSEP). Stimulation of the right and left median nerves was performed, and responses to point erb/C7 and contralateral somatosensory cortex (N19–P22) were analyzed. In 10 of these patients, SSEPs were recorded from the lumbar root entry area and the somatosensory cortex (P/N37) after stimulation of the right and left posterior tibial nerves. To date, 7/14 patients have undergone a VP shunt. On median nerve stimulation in the upper limbs, 12/14 patients had delayed P22 with respect to the erb point and an enlarged N19–P22 complex. In the lower limbs, the cortical response was abnormally delayed in 9/10 patients, and in 6/9 patients in which it was possible to calculate the CCT from the lumbar root to P/37, it was significantly prolonged. Clinical follow-up and CT were used to classify the responses as good, correct, or null. In total, 5/7 patients showed good clinical improvement. SSEPs improved in these 5 patients, but not in 2 who showed no clinical improvement. SSEP recordings can provide a useful noninvasive technique for assessing patients’ suitability for shunting in the NPH and following their course after the shunt, and also SSEP anomalies can also provide more information on the underlying pathophysiology of this condition. little-known clinic.

### 3.4 Motor evoked potentials

Gait disturbance is a cardinal symptom in NPH, and an improvement in it is useful for assessing outcome outcomes after shunt procedures.^[16]^ A study shows that motor evoked potentials and central motor conduction time (CMCT) have been examined in both the upper and lower limbs in patients with normal pressure hydrocephalus (NPH) to find a predictive factor for successful procedures of shunts as preoperative and postoperative evaluation.^[17]^ The study suggests that walking disorders are not the result of severe pyramidal tract dysfunction but probably involve sensorimotor integration leading to normal gait. In addition, CMCT measured with electromagnetic stimulation can assist in selecting patients who can benefit from shunting. The study did not provide electrophysiological evidence of upper limb involvement in normal-pressure hydrocephalus. Previous studies have shown a close association between frontal lobe dysfunction and gait disturbances in iNPH. A possible mechanism linking these impairments could be the modulation of corticospinal excitability. The aim was 2-fold: to determine whether iNPH influences corticospinal excitability, and assess changes in corticospinal excitability following placement of the ventricular shunt in relation to the clinical outcome. Twenty-three iNPH patients were examined using single-pulse and coupled transcranial magnetic stimulation of the motor area of the leg before and 1 month after ventricular shunt surgery. The corticospinal excitability parameters assessed were the resting motor threshold (rMT), motor evoked potential/M wave area ratio, CMCT, intracortical facilitation, and short intracortical inhibition (SICI). This study showed that iNPH influences corticospinal excitability, causing disinhibition of the motor cortex. The recovery of corticospinal excitability after ventricular shunt placement is related to clinical improvement. A significant reduction in SICI was associated with a decrease in rMT in patients with iNPH at the baseline evaluation. The positioning of the ventricular shunt resulted in a significant improvement in SICI and an increase in rMT in patients who markedly improved, but not in those who failed to improve. These findings support the view that impaired central motor conduction is responsible for gait disturbances in patients with iNPH.^[18]^

### 3.5 Electroencephalogram (EEG)

The significance of electroencephalography (EEG) in the evaluation of NPH has not often been studied. The general symptoms of iHPN can be improved after shunt operation. In patients treated with a VP shunt, Magneas et al and Matousek et al^[19]^ reported that the EEG tends to normalize with clinical improvement. However, all patients (iNPH) do not necessarily show clinical improvement after surgery. Before the shunt operation, a small amount of CSF (30–50 mL) is drained via lumbar puncture to predict the effect of the operation.^[20]^ The entire procedure is referred to as the CSF tap test. The EEG appears to be a useful tool for clinical evaluation and evaluation of brain functional changes induced by CSF drainage. Unlike fMRI and SPECT, which measure hemodynamic changes that occur in response to neuronal activity, neurophysiological techniques such as EEG and MEG directly measure brain electrical activity.^[21,22]^ In particular, dynamic postsynaptic activity in the cerebral cortex has high temporal resolution.^[23,24]^ For these properties, it is a simple and noninvasive examination that is widely used in clinical practice. Aoki et al^[25]^ aimed to identify a neurophysiological target marker of brain response to CSF touch in patients with iNPH. To detect cortical functional changes They examined the variant of normalized power (NPV) of EEG waves, calculated by neuronal activity topography analysis, as NPV is thought to reflect the phase instability of cortical electrical activity.^[26]^ They hypothesized that EEG phase instability is related to functional impairment and that NPV decreases and increases after CSF drainage in iNPH. This indicates that neuronal activity topography analysis is a sensitive and objective method for reliably detecting changes in cortical functions induced by CSF withdrawal in patients with iNPH. These results suggest that cortical functional recovery induced by CSF drainage is associated with changes in the VAN. More specifically, the improvement in walking time attributed to the CSF touch was correlated with an alpha NPV reduction in the supplementary motor area, and an alpha and beta NPV reduction decreased in the prefrontal cortex (PFC), especially in the anterior right PFC. In addition, recovery of gait status was delta-related and alpha NPV decreased in the left dorsal premotor area and attention recovery related to an alpha NPV decrease in the right dorsolateral PFC. These results demonstrate the importance of the involvement of the right anterior PFC and the premotor cortex in improving CSF-induced gait in iNPH patients. Aoki et al,^[27]^ later in another more recent study, aimed to distinguish shunt responders from nonresponders using only pre-CSF EEG data, that is, without performing CSF drainage, which would be significantly beneficial for iNPH patients as an alternative to classic procedures for predicting the outcome of the shunt operation; however, CSF drainage is an invasive procedure and presents risks of postprocedural infections such as headache and bleeding. They used the NPV of waves of EEG, and found that the NPV was significantly higher in the beta frequency band corresponding to the right fronto-temporo-occipital electrodes (Fp2, T4, and O2) in shunt responders than in nonresponders. Using these differences, they were able to correctly identify responders and nonresponders to the operation shunt with a positive predictive value of 80% and a negative predictive value of 88%. The results indicate that NPV may be useful in a noninvasive way to predict the clinical outcome of shunt surgery in patients with iNPH. In a recent study, Aoki et al,^[28]^ using the exact analysis of the components independent of low-resolution electromagnetic brain tomography (eLORETA-ICA) with EEG data, they evaluated the activities of 5 resting EEG networks (EEG-RSNs) in 58 iNPH patients before and after drainage of the CSF by lumbar puncture (tapping of the CSF). In addition, they assessed the correlations of changes in these 5 EEG-RSN activities with CSF-induced changes in iNPH symptoms. In addition, CSF induced changes in occipital alpha activity related to changes in postural sway and frontal lobe function. The results indicate the recruitment of cognitive networks in gait control and the involvement of alpha occipital activity in cognitive dysfunction in patients with iNPH. Based on these results, eLORETA-ICA with EEG data can be considered a non-invasive and useful tool for the detection of EEG-RSN activities and for understanding the neurophysiological mechanisms underlying this disease. Together, these results suggest that gait disturbance is believed to result from dysfunction of the cortico-striatal-thalamus-cortical network in the iNPH. However, the specific cortical networks involved in gait disturbance in this disease are yet to be clarified. Some authors have studied the correlation between the appearance of pressure waves and the level of sleep in pre- and postoperative patients with NPH. Seventeen patients were evaluated for suspected HPN disease, and 13 were treated with a VP shunt. Four cases were evaluated by pre- and postoperative polygraphic studies. In the nocturnal polygraphic study, in addition to EEG monitoring, intracranial pressure (ICP), electrooculogram, respiratory movement, and electromyography were monitored. It was observed that pressure waves occurred frequently, and as a result, the sleep level alternated frequently between the awake stage and stage 1. In practice, sleep levels are frequently interrupted by pressure waves and apneas. Such pathological states of sleep are indicative of nocturnal apnea syndrome.^[29]^ Furthermore, simultaneous polysomnography was performed to study the relationship of spontaneous ICP oscillations with different sleep phases.^[30]^ Intraventricular intracranial pressure was recorded continuously overnight in 16 patients with suspected hydrocephalus at normal pressure. The interpretation of data from continuous intracranial pressure monitoring (ICP) in patients with suspected NPH is controversial. Although ICP overnight monitoring is widely used for the diagnosis of NPH, the regulatory criteria are poorly defined. Krauss et al^[29]^ have shown that there is a relationship between the relative frequency, absolute amplitude, wavelength, and morphology of the B waves and the different sleep phases. Knowledge of the relationship between spontaneous oscillations of the ICP and different sleep phases can help establish physiological bases and alterations. In addition, polysomnography may be useful to avoid misinterpretation of ICP records due to variability related to the sleep stage.

### 3.6 The neuropsychological and psychiatric aspects in patients with normal pressure hydrocephalus (NPH)

Patients with NPH exhibit neuropsychiatric and neuropsychological symptoms. Assessment of these aspects is important for early and differential diagnosis; in fact, this pathology, characterized by cognitive dysfunction, psychiatric symptoms, and gait disturbance, is usually associated with dementia. Major cognitive impairment is related to frontal lobe symptoms, including psychomotor slowing, attention, working memory, and executive functions. Compared to Alzheimer disease (AD), symptoms of the frontal lobe are more compromised in NPH than in AD patients, while AD patients appear more compromised in memory and orientation.^[31]^ Several studies have shown that NPH patients perform cognitive improvement in multiple cognitive domains.^[32–35]^ Saito et al^[36]^ showed that memory, attention, language, executive function, and visuospatial abilities are more impaired in NPH patients than in patients with AD, whereas memory impairment in NPH patients was similar to that in AD patients. Tests that had the best evidence for the evaluation of NPH in the literature are presented in Table 1).^[37]^ Another study demonstrated that cognitive and neuropsychiatric symptoms in NPH may be associated with reduced volumes of subcortical regions; in particular, volume reductions of deep gray matter such as nucleus accumbens and caudate are associated with neuropsychological impairment and increased gravity of neuropsychiatric symptoms.^[38]^ Surgical treatment seems to be the best method for treating hydrocephalus. Several studies have demonstrated cognitive and neuropsychological changes after surgery. These findings suggest that shunt surgery is most sensitive for improving global cognition, learning, memory, and psychomotor speed.^[39,40,41]^ However, these results provided evidence for some cognitive-specific domains analyzed, such as the mini mental state examination, suggesting robust evidence for improved memory skills, fluency, and attentive processes.^[39]^ Solana et al,^[42]^ demonstrated that after shunting, an improvement in executive function was observed, and in mini mental state examination scores, this test represented the best predictor for the cognitive outcome. Another study observed that after surgery, NPH patients had increased mnestic abilities, and the Rey Auditory Verbal Learning Test was the neuropsychological test recommended to evaluate NPH before and after shunt because it was the test that demonstrated a significant improvement scores.^[43]^ Other studies showed discordant findings about the possible improvement of the cognitive performance postshunt, and they found that cognitive impairment before shunt surgery was associated with neurodegenerative disorder postshunt in follow-up evaluation.^[44,45]^

**Table 1:** Neuropsychological tests more used for evaluation of NPH.

Neuropsychological tests
Stroop Test
Digit Span
Rey Auditory Verbal Learning Test
Line Tracing
Trail Making Test A
Trail Making Test B
Word Fluency
Rey Osterrieth complex figure test simple reaction time
Wechsler memory scale Ten-word-list
Target reaction time
Finger Tapping

#### 3.6.1 Psychiatric aspects of NPH patients

NPH patients suffered from psychiatric symptoms in of 73.4%. The most common psychiatric symptoms associated with NPH are apathy (70.3%) and anxiety (25%).^[46]^ Studies have demonstrated that these disorders increase after surgery,^[47]^ and other studies have demonstrated that these disturbances could be reduced after shunt.^[48]^ Psychiatric symptoms are present in most patients with NPH,^[49,50]^ some patients develop symptoms with frontal dominance, aggressive behavior^[51]^ psychosis, and obsessive-compulsive disorder. Several studies demonstrated that it is possible to obtain an improvement of psychotic symptoms. The careful use of antidepressant and antipsychotic drugs and electroconvulsive therapy led to a partial improvement in these symptoms.^[52]^ Another study demonstrated the utility of the 2nd generation antipsychotics.^[51]^ Neuropsychiatric symptoms in patients with NPH were apathy, anxiety, and aggression; however, compared to AD there were no more prevalent symptoms, while other findings indicated a correlation between neuropsychiatric disorders and cognitive impairment and may depend on a common pathology of the frontal lobe.^[48]^ Studies have demonstrated that surgical treatment is useful for reducing psychiatric manifestations with a lumbo-peritoneal shunt.^[53]^ The literature findings suggested that disturbances such as obsessive-compulsive disorder were treated with neurosurgery, and Abbruzzese et al^[54]^ presented a case report that after surgery, the patient had an improvement in his compulsions and responded better to pharmacological therapy.

## 4 Discussion

This narrative review highlighted the need to exploit a complexity of methods to monitor the cognitive function and the effects that hydrocephalus has on the cerebral system. However, our results showed a discordance between what could be measured and what is actually made in clinical practice. In fact, there are no studies that univocally use the same clinical protocols and assessments. It is well known that neurosurgery is the most widely used practice for the resolution of NPH. A study used the health-related quality-of-life approach to evaluate important aspects of the wellness of NPH patients related to the outcome of CSF shunting. The results of this study are useful for clinicians to evaluate which patients could benefit from shunt surgery.^[55]^ A very important aspect to be considered concerns the monitoring of the effects that a hydrocephalus resorption can generate in the recovery of the patient. In fact, this review contribute not only to determine neurophysiological data neuropsychological and neuropsychiatric data that must be considered to identify this pathology, but a second objective, no less important, was to contribute to the creation of a clear and effective diagnostic protocol for early diagnosis and construct an appropriate intervention so that patients can improve their quality of life. Based on a complete review of the recent literature, we conclude that the early clinical NPH syndrome in the diagnostic process is the assessment of ventriculomegaly in brain imaging. NPH management remains a topic of discussion, marked by numerous controversies regarding the diagnostic and therapeutic strategies.^[20,27]^ An interventional point of view offers the possibility to have many therapeutic and surgical strategies, highlighting, in line with the purpose of our review, how the neurophysiological markers are essential to monitor the evolution of the disease. The confirmation of this disease is quite complex and there are not definitive tests or investigations that can accurately diagnose the condition^[31,33]^; therefore, each situation must be assessed individually in order to prevent incorrect diagnoses. In fact, cognitive aspects are often attributed to neurodegenerative diagnoses rather than considering the impairment as a cognitive marker of a hydrocephalus-related disorder. Therefore, cognitive assessment is also fundamental to develop therapeutic and rehabilitative interventions to promote an improvement of mental health and quality of life. Further studies are needed to study the differences in management and outcomes between the different etiologies of sNPH. Therefore, we can assume that neurophysiological methods are a very useful tool in the evaluation of brain functional changes induced by cerebrospinal drainage, with their simplicity and that they can be widely used to support a valid diagnosis of NPH.^[14,18]^ However, the prevalence of comorbidities is very high; neuropsychological assessment is not invasive and is an economic tool for objective cognitive and behavioral measurements. The results of this review are encouraging because this topic is not underestimated by the scientific community. However, future research should focus on the use of neurophysiological monitoring and diagnostic methods in order to find standardized clinical procedures.

## Author contributions

**Conceptualization:** Katia Micchia, Caterina Formica.

**Data curation:** Simona De Salvo, Nunzio Muscarà.

**Methodology:** Francesco Corallo.

**Supervision:** Simona De Salvo, Fabrizia Caminiti.

**Validation:** Placido Bramanti, Silvia Marino.

**Visualization:** Silvia Marino, Francesco Corallo.
